# Mental Distress, Fatigue and Executive Function in Adult Survivors of Childhood Leukemia and Non-Hodgkin Lymphoma

**DOI:** 10.3390/curroncol33070397

**Published:** 2026-07-01

**Authors:** Anna R. Franzén, Jan Stubberud, Torstein B. Rø, Stian Lydersen, Kaja S. Egset, Ellen Ruud, Siri Weider, Mary-Elizabeth Eilertsen, Anne Mari Sund, Trude Reinfjell, Magnus A. Hjort

**Affiliations:** 1 Department of Clinical and Molecular Medicine, Norwegian University of Science and Technology, 7491 Trondheim, Norway; torstein.ro@ntnu.no (T.B.R.); magnus.a.hjort@ntnu.no (M.A.H.); 2 Department of Psychology, University of Oslo, 0373 Oslo, Norway; j.e.stubberud@psykologi.uio.no; 3 Department of Research, Lovisenberg Diaconal Hospital, 0440 Oslo, Norway; 4 Children’s Clinic, St. Olavs Hospital, Trondheim University Hospital, 7006 Trondheim, Norway; 5 Regional Center for Child and Youth Mental Health and Child Welfare, Norwegian University of Science and Technology, 7030 Trondheim, Norway; stian.lydersen@ntnu.no (S.L.); anne.m.sund@ntnu.no (A.M.S.); 6 Department of Psychology, Norwegian University of Science and Technology, 7491 Trondheim, Norway; kaja.solland.egset@ntnu.no (K.S.E.); siri.weider@ntnu.no (S.W.); trude.reinfjell@ntnu.no (T.R.); 7 Division for Pediatric and Adolescent Medicine, Oslo University Hospital, 0424 Oslo, Norway; elruud@ous-hf.no; 8 Institute of Clinical Medicine, University of Oslo, 0372 Oslo, Norway; 9 Department of Public Health and Nursing, Center for Health Promotion Research, Norwegian University of Science and Technology, 7491 Trondheim, Norway; mary-elizabeth.eilertsen@ntnu.no

**Keywords:** childhood cancer, survivors, mental distress, fatigue, executive function

## Abstract

Survivors of childhood acute lymphoblastic leukemia (ALL), acute myeloid leukemia (AML), and non-Hodgkin lymphoma (NHL) are at risk of developing long-term adverse effects after survival. This study examined observed proportions of perceived mental distress, fatigue, and executive function (EF) impairment in adult childhood cancer survivors (CCSs) of ALL, AML, and NHL. Secondly, it examined the association between perceived EF impairment and mental distress or fatigue. Participants (*n* = 132; 57% female) were recruited from two major Norwegian hospitals. Self-report questionnaires included the Behavior Rating Inventory of Executive Function, Adult Version, the Hopkins Symptom Checklist-25, and the Fatigue Severity Scale. Proportions exceeding established clinical thresholds were calculated, and groups were compared using Pearson’s chi-squared test and Newcombe confidence intervals. Overall, 49% and 41% of participants met the clinical thresholds for depression and anxiety; 43% for fatigue; and 28% for EF impairment. Perceived EF impairment was significantly associated with mental distress and fatigue. Mental distress, fatigue, and EF impairment are commonly reported and distressing late effects among CCSs of ALL, AML, and NHL. Follow-up care focusing on neurocognitive and psychological outcomes is important for the long-term functioning and well-being of this survivor group. Targeted neurocognitive rehabilitation may represent a key component of follow-up care.

## Simple Summary

Advances in cancer treatment have increased survival rates for childhood leukemia and lymphoma, resulting in a growing population of survivors at risk of long-term sequelae. Beyond physical late effects, survivors may report mental distress, fatigue, and neurocognitive difficulties, including impairment in executive functioning, which affect participation in education and work, social functioning, and quality of life. The relationship between these symptoms remains insufficiently studied in survivors of childhood leukemia and lymphoma. In the present study, we identified substantial levels of perceived mental distress, fatigue, and executive function impairment in Norwegian adult survivors. Perceived executive functioning impairment was significantly associated with mental distress and fatigue. Longitudinal studies are needed to further clarify the temporal relationships between these long-term sequelae. This knowledge can inform targeted interventions aimed at improving daily functioning and long-term psychosocial adaptation. Neurocognitive rehabilitation may represent a promising component of survivorship care to support quality of life in this population.

## 1. Introduction

The prognosis for childhood cancer has improved over the past few decades, driven by the advancement of effective, risk-stratified treatment protocols [1]. Today, five-year survival rates for childhood leukemia and lymphoma combined exceed 90% [2], contributing to a growing population of childhood cancer survivors (CCSs). Acute lymphoblastic leukemia (ALL), acute myeloid leukemia (AML), and non-Hodgkin lymphoma (NHL) constitute approximately 30% of childhood cancer cases in Norway [2]. Treatment strategies for ALL, AML, and NHL have evolved toward multimodal, more personalized, and targeted approaches, aiming to maintain high cure rates while reducing long-term toxicity [3]. However, many survivors still experience long-term late effects after cancer treatment [4]. Mental distress (anxiety and depression), fatigue, and neurocognitive impairment are among the most frequently reported late effects in CCSs and are each associated with impaired daily functioning and reduced quality of life [5,6,7,8,9].

Executive function (EF) refers to a set of higher-order neurocognitive processes essential for goal-directed behavior, including concentration, selective attention, inhibition, mental set-shifting, and creativity, crucial for regulating thoughts, emotions, and actions in everyday life [10]. CCSs have been shown to be more vulnerable to EF impairment compared with healthy controls [7,11], potentially compromising educational attainment, occupational functioning, and independence [1]. Moreover, EF impairment has been associated with increased symptoms of mental distress and fatigue in several clinical populations, including adult cancer survivors [12,13] and children with chronic health conditions or brain injuries [14,15].

Previous studies on CCSs have linked neurocognitive impairment with mental distress, fatigue, and reduced quality of life. However, research has focused on general neurocognitive complaints, treatment-related risk factors, or pediatric CCS populations [16,17,18]. Less is known about how perceived EF impairment specifically relates to mental distress and fatigue in adult survivors of childhood ALL, AML, and NHL. To our knowledge, studies examining these associations using comprehensive self-report measures with established clinical thresholds are lacking.

### Aims

The present study examined the proportions of perceived mental distress, fatigue, and EF impairment in adult CCSs of ALL, AML, and NHL in Norway. In addition, the study explored the associations between perceived EF impairment and mental distress or fatigue.

## 2. Materials and Methods

### 2.1. Study Design

This cross-sectional study investigated the associations between mental distress, fatigue, and EF impairment among adult survivors of childhood ALL, AML, and NHL.

### 2.2. Participants and Procedures

The study population was identified through the Cancer Registry of Norway and consisted of individuals diagnosed with childhood ALL, AML, or NHL between 1980 and 2017, who received treatment at Oslo University Hospital or St. Olavs Hospital in Norway. Participants were included based on the following criteria: age 18–40 years, ≥5 years post-diagnosis, completion of cancer treatment at the time of study participation, and survival through 2020–2021. Individuals with ADHD, motor or sensory impairment, severe psychiatric disease, substance abuse, suicidal ideation, Down syndrome, or premorbid central nervous system (CNS) disease or injury were excluded. Participants were included after receiving an invitation letter and providing written informed consent. Of the 479 invited, 143 agreed to participate (30% response rate). This study was part of a larger clinical trial (clinicaltrials.gov: NCT04541056) that offered neurocognitive rehabilitation to participants reporting EF impairment. Neurocognitive sequelae were not a prerequisite for inclusion in the present study. This study was approved by the Regional Committee for Medical Ethics Central Norway (REK 2018/1810).

### 2.3. Measurements

Demographic data, including education, employment, financial status, and adverse life events, were gathered through semi-structured interviews. Clinical data, including diagnosis, treatment type, and relapse history, were obtained from hospital records. The study outcomes included assessment of mental distress, fatigue, and EF impairment using validated self-reports.

### 2.4. Self-Report Questionnaires

The Hopkins Symptom Checklist (HSCL-25) was used to measure mental distress symptoms on a 25-item, four-point Likert scale (1 = not at all, 2 = a little, 3 = quite a bit, 4 = extremely). The questionnaire contains ten items for anxiety symptoms and fifteen for depression. Participants rate according to agreement on experiencing symptoms such as being nervous or anxious, experiencing palpitations, feeling low interest in things, or worrying too much. The threshold for clinically significant anxiety or depression is a mean score greater than or equal to 1.75 (cut-off ≥ 1.75) [19].

The Fatigue Severity Scale (FSS) was used to assess fatigue severity and its impact on daily functioning. Participants respond to nine statements using a seven-point Likert scale (1 = strong disagreement, 7 = strong agreement) such as “I am easily fatigued” and “fatigue interferes with my work, family, or social life”. The threshold for clinically significant severe fatigue is a mean value greater than or equal to 5 [20].

The Behavior Rating Inventory of Executive Function, Adult (BRIEF-A) [21] was used to assess everyday EF. Participants rated 75 items on a three-point Likert scale (1 = never, 2 = sometimes, 3 = often) with statements such as “I have a short attention span” and “I easily forget instructions”. The BRIEF-A includes nine subscales, which contribute to three index scores: the Global Executive Composite (GEC), an overall measure of EF; the Metacognitive Index (MI); and the Behavioral Regulation Index (BRI). The MI comprises subscales Initiate, Working Memory, Plan/Organize, Task Monitor, and Organization of Materials, whereas the BRI comprises Inhibit, Shift, Emotional Control, and Self-Monitor. Raw scores were converted to T-scores, and a T-score ≥ 65 (1.5 SD above the mean) indicated clinically significant EF impairment on the BRIEF-A index scores (GEC, MI, or BRI).

### 2.5. Statistical Analysis

For each scale, total scores were calculated by multiplying the mean item score by the number of items. Missing data accounted for 0–0.0017% of total items, and missing values were handled using available case analysis; that is, each analysis included participants with available data on the relevant variables. Continuous variables were categorized following practices used in previous research, ensuring comparability of results [22]. The variables for BRIEF-A, HSCL anxiety, HSCL depression, and FSS were dichotomized according to the established thresholds defined above. Descriptive statistics are reported as means and standard deviation (SD) for scale variables, and counts (proportions) for categorical variables. The primary association analyses were conducted using the BRIEF-A index scores (GEC, MI, and BRI), whereas the nine BRIEF-A subscales were examined descriptively. Proportions were compared using the Pearson chi-squared test and Newcombe hybrid score confidence intervals for the difference between proportions. Next, we used logistic regression with depression, anxiety, or fatigue as the dependent variable, and EF impairment as the dichotomous independent variable. This was done unadjusted, adjusted for one adjustment variable at a time, and finally including the interaction between the adjustment variable and EF impairment. Adjustment variables included demographic, treatment, and medical factors. Statistical analysis was performed using IBM-SPSS version 29 (SPSS, Inc., Chicago, IL, USA).

## 3. Results

A total of 132 participants (M_age_ = 28.1, SD = 6.5, 56.8% female) were included. Of these, 112 were diagnosed with ALL, 4 with AML, and 16 with NHL. The predominant treatment protocols for ALL survivors were NOPHO-ALL 1992, 2000, and 2008; for AML survivors, various NOPHO-AML protocols; and for NHL survivors, the NHL-BFM or NOPHO-NHL protocols. For ALL, participants were categorized according to their treatment approach into high-risk (*n* = 24, 22%), non-high-risk (*n* = 63, 58%), and unspecified risk groups (*n* = 22, 20%). Further demographic, treatment, and medical characteristics are provided in Table 1.

### 3.1. Perceived EF Impairment, Mental Distress, and Fatigue

Overall, 49% and 41% of participants met the clinical thresholds for depression and anxiety, respectively, and 43% met the clinical thresholds for severe fatigue. For EF impairment, 28% met the clinical threshold for the GEC, 34% for the MI, and 15% for the BRI; descriptive BRIEF-A subscale scores are presented in Table 2.

The proportions of participants scoring above the clinical threshold for BRIEF-A (GEC, MI, BRI), HSCL, and FSS differed by gender, employment status, and financial status (Table 3). Females were more likely to report anxiety and fatigue. Unemployment was associated with depression, fatigue, and EF impairment, while self-perceived low income was associated with EF impairment, anxiety, and depression. No significant associations were identified for medical or treatment-related factors.

### 3.2. Association Between EF Impairment and Mental Distress or Fatigue

Comparisons of proportions showed significant associations between EF impairment (GEC, MI, and BRI) and mental distress or fatigue. There were associations between GEC and higher rates of anxiety (risk difference = 49%, 95% CI [30 to 62], *p* < 0.001), depression (risk difference = 49%, 95% CI [31 to 61], *p* < 0.001), and fatigue (risk difference = 45%, 95% CI [27 to 59], *p* < 0.001) (Table 4). Adjustments in logistic regression for demographic, medical, and treatment-related variables did not substantially change the associations between EF impairment and anxiety, depression, or fatigue.

## 4. Discussion

The present study aimed to examine proportions of perceived mental distress, fatigue, and EF impairment as well as to explore the associations between EF impairment and mental distress or fatigue in adult survivors of ALL, AML, NHL. In this sample, 49% and 41% met the clinical thresholds for depression and anxiety respectively, and 43% met the clinical thresholds for severe fatigue. These proportions exceed those reported in previous studies, where fatigue has been observed in 26–30% of CCSs [8,17] and mental distress in 15–27% [23,24]. The discrepancy may be attributed to differences in sample characteristics, as larger studies often encompass more diverse demographics, varying treatment exposure, and follow-up durations. Furthermore, assessment methods might capture a broader spectrum of subjective impairment. This cohort presents long-term adult survivors treated with Nordic protocols and may reflect a unique subset of survivors with specific vulnerabilities.

Using the BRIEF-A GEC as the overall composite measure of EF impairment, 28% scored above the clinical threshold. This proportion is consistent with findings from a previous Norwegian study [7] and notably higher than rates observed in healthy controls, where fewer than 6% scored above the clinical threshold [25]. However, the observed proportions of EF impairment in the present study should be interpreted with caution and not as a population prevalence estimate, as recruitment from a larger neurocognitive rehabilitation trial may have enriched the sample for individuals with perceived neurocognitive impairment. Furthermore, deficits were more pronounced in metacognitive EF compared to behavioral aspects, with 34% scoring above the clinical threshold on the MI and 15% on the BRI. Similar patterns have been observed in adult survivors of childhood brain tumors [11] and healthy controls [25]. Descriptively, the highest mean scores were observed for the Working Memory and Initiate subscales, whereas the lowest scores were observed for Organization of Materials and Self-Monitor. However, these findings should be interpreted cautiously, as analyses at the subscale level were not prespecified. Further analyses of the BRIEF-A subscales may provide insight into specific EF domains and their contribution to mental distress and fatigue.

Furthermore, we found that survivors with perceived EF impairment following cancer and its treatment were at elevated risk of mental distress. EF plays a central role in emotion regulation, stress management, and adaptive coping [10]. Impairment in these processes such as inhibitory control and neurocognitive flexibility may make it more challenging to manage negative thoughts and emotions, potentially increasing vulnerability to anxiety and depression [26,27]. We observed that impairments in both metacognitive and behavioral aspects of EF were associated with mental distress, although the strongest associations were identified for the BRI, reflecting impairment in emotional regulation and inhibition. At the same time, mental distress has also been linked to emotion regulation processes [28], and adaptive regulatory strategies have been shown to support EF performance [29]. Reverse causation should also be considered, as symptoms of mental distress may contribute to perceived EF impairment [28]. The findings further suggest that the BRIEF-A may reflect a broader combination of neurocognitive and psychological factors, rather than serving solely as a measure of everyday EF [30].

Next, we found that survivors with perceived EF impairment were at increased risk of fatigue. This underscores the multifaceted nature of fatigue in CCSs, influenced by various factors that extend beyond mere physical outcomes. EF impairment may contribute to fatigue by reducing the capacity to manage stress, regulate emotions, and sustain attention during daily tasks [10]. As a result, academic, occupational, and social demands may require greater executive effort, potentially increasing the fatigue burden [31]. Previous research has highlighted fatigue as a core aspect of the control mechanisms that support and sustain goal-directed behavior [32] and as a potential contributor to poorer neurocognitive functioning in CCSs [33]. Furthermore, fatigue has been associated with poorer mental well-being [31], and depression-related mechanisms, such as rumination, have been shown to increase fatigue and reduce resilience among survivors [34].

Together, these findings suggest that EF impairment, mental distress, and fatigue may be closely interconnected through shared self-regulatory and emotional processes. This highlights the potential importance of EF in understanding and addressing complex survivorship challenges. Rehabilitation intervention studies targeting these domains remain limited among CCSs, with a particular knowledge gap regarding long-term adult survivors [35]. While the cross-sectional design precludes conclusions regarding causality, the present findings may help identify promising targets for future intervention studies. They further suggest that interventions focusing solely on neurocognitive outcomes may be insufficient. At the same time, future studies should investigate whether improvements in EF are accompanied by improvements in mental well-being and reductions in fatigue burden.

Several demographic outcomes were associated with experiencing mental distress, fatigue, and EF impairment. Gender differences emerged, with females more likely to report anxiety and fatigue, a well-documented trend in the literature [8,36]. Unemployment was associated with EF impairment, fatigue, and depression, while low income was associated with EF impairment, anxiety, and depression. CCSs are at a higher risk of experiencing socioeconomic challenges [37], and similar associations have been shown in previous research [8,36]. EF impairment, mental distress, and fatigue may not only limit employment opportunities, but also exacerbate financial strain, amplifying stress that profoundly affects overall functioning. While previous studies have linked time since diagnosis and younger age at diagnosis to poorer neurocognitive outcomes [4], the present study found no significant associations between either of these factors and EF impairment. Instead, these findings support research highlighting stronger associations between demographic factors [38]. This underscores the importance of addressing socioeconomic and demographic factors in post-treatment care for childhood cancer survivors.

A strength of this study is the diversity of the sample, which was drawn from two of Norway’s largest childhood hospitals. Although potential regional differences were not examined, both centers operate within a similar national healthcare framework and sociocultural context. Given that odds ratio can overestimate associations when event rates are high [39], presenting risk differences provides a more accurate measure in this study. Several limitations should be considered. First, while data categorization may have limited findings [22], it was applied to enhance comparability across variables. A low response rate further limits the generalizability of the results, and the absence of information on non-respondents precludes evaluation of potential selection bias. The healthiest survivors and those experiencing greater impairment may have been more motivated to participate, and recruitment may have enriched the sample with individuals experiencing EF impairment. The limited number of AML and NHL survivors restricted diagnosis-specific analyses, and the findings should be interpreted primarily at the overall group level, likely reflecting patterns driven predominantly by ALL survivors. Furthermore, the lack of a control group limits direct comparisons with normative populations. Although self-report measures are valuable for assessing daily EF, they are prone to information bias [40], particularly in a population where subjective perception may vary. Incorporating objective measures, such as neurocognitive tests, and clinical interviews would provide a more in-depth assessment. Furthermore, no study-specific power calculation was performed for the present study. Although the study aims and statistical analyses were prespecified, the findings should be interpreted with caution and in light of the confidence intervals, particularly for smaller subgroups. Finally, longitudinal designs are needed to clarify causal relationships.

## 5. Conclusions

This study highlights substantial levels of perceived mental distress, fatigue, and EF impairment in long-term adult survivors of childhood ALL, AML, and NHL. Secondly, perceived EF impairment was significantly associated with mental distress and fatigue. Future longitudinal studies including measures of mental distress, fatigue, and EF impairment are needed to investigate the temporal relationships among these constructs. Furthermore, intervention studies should evaluate whether improving EF may contribute to improved mental well-being, reduced fatigue, and enhanced quality of life among CCSs of leukemia and lymphoma.

## Figures and Tables

**Table 1:** Characteristics of the 132 participants.

	*N* (%)	Mean (SD)	Min.–Max.
Gender	Female: 75 (57)		
Age at inclusion (years)	18–25: 50 (38) 26–33: 49 (37) 34–40: 33 (25)	28.1 (7)	18–40
Type of malignancy	ALL: 112 (85) AML: 4 (3) NHL: 16 (12)		
Hospital	Oslo University Hospital: 99 (75) St. Olavs Hospital: 33 (25)		
Age at diagnosis	1–6: 63 (48) 7–12: 34 (26) 13–18: 29 (22) Unknown: 6 (4)	7.8 (5)	1–18
Years since last anti-cancer treatment	0–10: 18 (14) 11–21: 58 (44) 22–32: 43 (32) Unknown: 13 (10)	17.7 (7)	0–32
ALL high-risk treatment protocol (***n*** = 109)	Yes: 24 (22) No: 63 (58) Unspecified according to risk stratification: 22 (20)		
Radiotherapy (craniospinal)	Yes: 13 (10) No: 114 (86) Unknown: 5 (4)		
Relapse	Yes: 13 (10) No: 114 (86) Unknown: 5 (4)		
Education years		14.8 (2)	9–20
Main occupation	Employment > 50%: 54 (41) Employment ≤ 50%: 6 (5) Unemployment: 19 (14) Student: 38 (29) Other/Unknown: 15 (11)		
Financial status	Above average: 25 (19) Average: 76 (58) Below average: 29 (22) Unknown: 2 (1)		

ALL: acute lymphoblastic leukemia; AML: acute myeloid leukemia; NHL: non-Hodgkin lymphoma; “unemployment” represents participants without a primary occupation due to being unemployed, receiving disability benefit, or being on sick leave. “Educational years” refers to the number of years of formal education completed by the participants. “Financial status” refers to participants’ self-assessment of their financial situation.

**Table 2:** Mean scores and proportions of participants scoring above the clinical threshold for the Behavior Rating Inventory of Executive Function, Adult (BRIEF-A), including Global Executive Composite (GEC), Metacognitive Index (MI), and Behavioral Regulation Index (BRI), the Hopkins Symptom Checklist (HSCL), and the Fatigue Severity Scale (FSS).

	All Participants (*n* = 132)				Above Cut-Off, *N* (%)		
	All Mean (SD)	*Female*	Male	Min.–Max.	All	Female	Male
**BRIEF-A (GEC) (cut-off ≥ 65)**	**57.5 (11.3)**	**59.0 (12.1)**	**55.7 (10.0)**	**35** **–** **83**	**37 (28)**	**26 (35)**	**11 (19)**
**BRIEF-A (MI) (cut-off ≥ 65)**	**59.4 (11.2)**	**60.2 (11.9)**	**58.4 (10.2)**	**37** **–** **83**	**45 (34)**	**30 (40)**	**15 (26)**
Initiate	60.8 (12.8)	60.5 (13.2)	61.1 (12.4)	37–94			
Working memory	63.5 (12.7)	65.4 (13.5)	61.0 (11.1)	39–91			
Plan/organize	57.4 (11.2)	57.6 (11.9)	57.1 (10.4)	38–86			
Task monitor	57.7 (10.8)	57.6 (11.6)	57.9 (9.8)	36–86			
Organization of materials	51.4 (10.5)	52.3 (10.6)	50.1 (10.4)	36–78			
**BRIEF-A (BRI) (cut-off ≥ 65)**	**53.8 (11.3)**	**55.9 (12.0)**	**51.1 (9.9)**	**35** **–** **83**	**20 (15)**	**15 (20)**	**5 (9)**
Inhibit	52.4 (9.6)	52.6 (10.0)	52.1 (9.0)	36–78			
Shift	56.5 (12.8)	58.1 (12.9)	54.5 (12.5)	8–87			
Emotional control	53.5 (13.0)	57.3 (13.4)	48.6 (10.8)	38–83			
Self-monitor	49.2 (10.4)	48.9 (10.3)	49.5 (10.7)	37–86			
**Hopkins Symptom Checklist, HSCL-25 Anxiety (cut-off ≥ 1.75)**	**1.7 (0.5)**	**1.8 (0.6)**	**1.6 (0.4)**	**1.0** **–** **3.6**	**54 (41)**	**37 (49)**	**17 (30)**
**Hopkins Symptom Checklist, HSCL-25 Depression (cut-off ≥ 1.75)**	**1.9 (0.6)**	**2.0 (0.7)**	**1.8 (0.6)**	**1.0** **–** **3.7**	**64 (49)**	**40 (53)**	**24 (42)**
**FSS: Fatigue Severity Scale (cut-off ≥ 5)**	**4.4 (1.5)**	**4.7 (1.6)**	**4.0 (1.4)**	**1.3** **–** **7.0**	**57 (43)**	**39 (52)**	**18 (32)**

**Table 3:** Proportions over cut-off for BRIEF-A (GEC), HSCL, and FSS and risk difference (RD) based on demographic, medical and treatment-related factors.

**Gender (*n* = 132)**													
Index	Males (*n* = 57)						Females (*n* = 75)						RD for females (95% CI), *p*-value
GEC	11/57 (19%)						26/75 (35%)						15% (0.1% to 29%), 0.05
HSCL anxiety	17/57 (30%)						37/75 (49%)						19% (3% to 35%), 0.019
HSCL depression	24/57 (42%)						40/75 (53%)						11% (6% to 27%), 0.20
FSS	18/57 (32%)						39/75 (52%)						20% (3% to 36%), 0.019
**Age at participation (*n* = 132)**													
**Index**	18–25 (*n* = 50)				26–33 (*n* = 49)				34–40 (*n* = 33)				RD for age 34–40 compared to age 18–25 (95% CI), *p*-value
GEC	12/50 (24%)				11/49 (22%)				14/33 (42%)				18% (14% to 33%), 0.077
HSCL anxiety	18/50 (36%)				20/49 (41%)				16/33 (49%)				13% (9% to 33%), 0.26
HSCL depression	23/50 (46%)				25/49 (51%)				16/33 (49%)				3% (19% to 23%), 0.82
FSS	19/50 (38%)				21/49 (43%)				17/33 (52%)				14% (8% to 34%), 0.22
**Age at diagnosis (*n* = 126)**													
Index	1–6				7–12				13–18				RD for 13–18 compared to age 1–6 (95% CI), *p*-value
GEC	18/63 (29%)				10/34 (29%)				8/29 (28%)				−1% (20% to 19%), 0.92
HSCL anxiety	28/63 (44%)				14/34 (41%)				10/29 (35%)				−10% (12% to 29%), 0.34
HSCL depression	28/63 (44%)				19/34 (56%)				14/29 (48%)				4% (17% to 25%), 0.73
FSS	26/63 (41%)				15/34 (44%)				13/29 (45%)				4% (17% to 25%), 0.75
**Year since last anti-cancer treatment (*n* = 119)**													
Index	0–10				11–21				22–32				RD for 22–32 years compared to 0–10 years (95% CI), *p*-value
GEC	4/18 (22%)				18/71 (25%)				15/43 (35%)				13% (14% to 33%), 0.33
HSCL anxiety	6/18 (33%)				30/71 (42%)				18/43 (42%)				9% (18% to 31%), 0.53
HSCL depression	10/18 (56%)				33/71 (47%)				21/43 (49%)				−7% (19% to 31%), 0.63
FSS	7/18 (39%)				30/71 (42%)				20/43 (47%)				8% (19% to 31%), 0.56
**Risk group (ALL) (*n* = 87)**													
Index	High-risk						Non-high-risk						RD for non-high-risk (95% CI), *p*-value
GEC	19/63 (30%)						5/24 (21%)						−9% (13% to 26%), 0.38
HSCL anxiety	29/63 (46%)						6/24 (25%)						−21% (21% to 39%), 0.74
HSCL depression	32/63 (51%)						11/24 (46%)						−5% (18% to 27%), 0.68
FSS	31/63 (49%)						9/24 (38%)						−12% (11% to 32%), 0.33
**Radiotherapy (*n* = 127)**													
Index	No radiotherapy						Radiotherapy						RD for radiotherapy (95% CI), *p*-value
GEC	32/114 (28%)						4/13 (31%)						3% (17% to 31%), 0.84
HSCL anxiety	46/114 (40%)						5/13 (39%)						−1% (26% to 25%), 0.86
HSCL depression	55/114 (48%)						7/13 (54%)						6% (21% to 30%), 0.70
FSS	48/114 (42%)						7/13 (54%)						12% (15% to 36%), 0.42
**Relapse (*n* = 127)**													
Index	No relapse						Relapse						RD for relapse (95% CI), *p*-value
GEC	33/114 (29%)						3/13 (23%)						−6% (22% to 23%), 0.66
HSCL anxiety	48/114 (42%)						4/13 (31%)						−11% (17% to 32%), 0.43
HSCL depression	55/114 (48%)						6/13 (46%)						−2% (24% to 27%), 0.89
FSS	46/114 (40%)						8/13 (62%)						21% (6% to 44%), 0.14
**Educational years (*n* = 132)**													
Index	9–12				13–16				17–30				RD for 17–30 years compared to 9–12 (95% CI), *p*-value
GEC	8/27 (30%)				19/72 (26%)				10/33 (30%)				0% (22% to 23%), 0.96
HSCL anxiety	13/27 (48%)				30/72 (42%)				11/33 (33%)				−15% (10% to 37%), 0.24
HSCL depression	15/27 (56%)				32/72 (44%)				17/33 (52%)				−4% (20% to 26%), 0.32
FSS	16/27 (59%)				27/72 (38%)				14/33 (42%)				−17% (8% to 39%), 0.19
**Financial status (*n* = 130)**													
Index	Below average				Average				Above average				RD for above average (95% CI), *p*-value
GEC	11/29 (38%)				23/76 (30%)				3/25 (12%)				−26% (2% to 46%), 0.030
HSCL anxiety	16/29 (55%)				31/76 (41%)				7/25 (28%)				−27% (1% to 49%), 0.044
HSCL depression	21/29 (72%)				33/76 (43%)				10/25 (40%)				−32% (6% to 54%), 0.016
FSS	16/29 (55%)				29/76 (38%)				12/25 (48%)				−7% (18% to 32%), 0.60
**Occupation (*n* = 117)**													
Index	Employed >50%			Employed ≤50%			Un- employed			Student			RD for unemployment compared to employed >50% (95% CI), *p*-value
GEC	10/54 (19%)			2/6 (33%)			12/19 (63%)			9/38 (24%)			45% (19% to 64%), <0.010
HSCL anxiety	20/54 (37%)			3/6 (50%)			11/19 (58%)			13/38 (34%)			21% (5% to 45%), 0.11
HSCL depression	22/54 (41%)			3/6 (50%)			13/19 (68%)			20/38 (53%)			28% (16% to 48%), 0.038
FSS	22/54 (41%)			1/6 (17%)			14/19 (74%)			12/38 (32%)			33% (7% to 52%), 0.013
													RD for unemployment compared to student (95% CI), *p*-value
													40% (13% to 60%), 0.04
													24% (3% to 47%), 0.09
													16% (11% to 38%), 0.26
													42% (15% to 61%), <0.010

Table results presented were based on prior research and their relevance to the study hypothesis.

**Table 4:** Perceived anxiety, depression, or fatigue, for individuals without or with EF impairment (*n* = 132).

**HSCL Anxiety ≥ 1.75**			
**EF impairment index**	**EF impairment**		**Risk difference** **Estimate (95% CI), *p*-value**
	**No**	**Yes**	
GEC	26/95 (27%)	28/37 (76%)	49% (30% to 62%), <0.001
MI	25/87 (29%)	29/45 (64%)	39% (18% to 51%), <0.001
BRI	36/112 (32%)	18/20 (90%)	58% (36% to 60%), <0.001
**HSCL depression ≥ 1.75**			
**EF impairment index**	**EF impairment**		**Risk difference** **Estimate (95% CI), *p*-value**
	**No**	**Yes**	
GEC	33/95 (35%)	31/37 (85%)	49% (31% to 61%), <0.001
MI	29/87 (33%)	35/45 (78%)	45% (27% to 58%), <0.001
BRI	46/112 (41%)	18/20 (90%)	49% (27% to 60%), <0.001
**HSCL fatigue ≥ 5**			
**EF impairment index**	**EF impairment**		**Risk difference** **Estimate (95% CI), *p*-value**
	**No**	**Yes**	
GEC	29/95 (31%)	28/37 (76%)	45% (27% to 59%), <0.001
MI	25/87 (29%)	32/45 (71%)	42% (25% to 56%), <0.001
BRI	40/112 (36%)	17/20 (85%)	49% (26% to 62%), <0.001

Note: For example, among the individuals with GEC impairment, 28/37 (76%) report HSCL anxiety above the clinical threshold.

## Data Availability

The data supporting the findings of this study are not openly available due to policy restrictions and are available from the corresponding author upon reasonable request.
